# Iterative Refinement of Transmission Map for Stereo Image Defogging Using a Dual Camera Sensor

**DOI:** 10.3390/s17122861

**Published:** 2017-12-09

**Authors:** Heegwang Kim, Jinho Park, Hasil Park, Joonki Paik

**Affiliations:** Department of Image, Chung-Ang University, 84 Heukseok-ro, Dongjak-gu, Seoul 06974, Korea; heegwang27@gmail.com (H.K.); dkskzmffps@gmail.com (J.P.); hahaha2470@cau.ac.kr (H.P.)

**Keywords:** image enhancement, image restoration, stereo image processing, dehazing, defogging

## Abstract

Recently, the stereo imaging-based image enhancement approach has attracted increasing attention in the field of video analysis. This paper presents a dual camera-based stereo image defogging algorithm. Optical flow is first estimated from the stereo foggy image pair, and the initial disparity map is generated from the estimated optical flow. Next, an initial transmission map is generated using the initial disparity map. Atmospheric light is then estimated using the color line theory. The defogged result is finally reconstructed using the estimated transmission map and atmospheric light. The proposed method can refine the transmission map iteratively. Experimental results show that the proposed method can successfully remove fog without color distortion. The proposed method can be used as a pre-processing step for an outdoor video analysis system and a high-end smartphone with a dual camera system.

## 1. Introduction

Image analysis using multiple images has recently attracted growing attention in the fields of autonomous driving, unmanned surveillance cameras, and drone imaging service. It is important to acquire additional depth information as well as high-quality images in many sophisticated image analysis applications. Another market-leading application is the dual camera in a smartphone. The proposed stereo image-based defogging algorithm can be applied to an asymmetric dual camera system in a smartphone with a proper geometric transformation to improve the visibility of the outdoor foggy scene acquired by a smartphone. Specifically, fog component in the atmosphere decreases contrast, and as a result, makes extracting features or recognizing objects in image analysis difficult. Therefore, an image enhancement method to reduce the fog component is important to increase the reliability of the image analysis system.

Fog particles absorb and scatter the light reflected from the object and then transmitted to the camera. They also distort the original color and edge in a random manner. The amount of atmospheric distortion increases with the distance between a scene point and the camera. This phenomenon can be quantified using a transmission coefficient at each pixel. For this reason, the degraded foggy image is modeled as a combination of the original reflectance of the scene, the atmospheric component, and the transmission coefficient in a pixel-wise manner. Because of its importance in various image analysis applications, the image defogging problem has been intensively studied in the field of image processing and computer vision.

Narasimhan et al. corrected color distortion by estimating the distribution of fog according to the distance [1,2]. They acquired multiple images of the same scene under different weather conditions to construct a scene structure. Shwartz et al. and Schechner et al. proposed a defogging method by measuring the distribution of fog using two different polarized filters in the same scene [3,4]. These methods can successfully remove the fog component using a physically reasonable degradation model at the cost of inconvenience to acquire multiple images of the same scene.

To solve these problems, various single image defogging methods were proposed. Tan proposed a defogging method using the characteristics that a fog-free image has higher contrast than foggy images and color distortion caused by fog increases proportionally to the distance from the camera [5]. Based on these two characteristics, the Markov random field model was estimated and a fog-free image was obtained by maximizing the local contrast at the cost of contrast saturation and halo effect. Fattal removed fog using the property that the surface reflectance of the object is constant and the transmission depends on the density and depth information fog [6]. However, it is difficult to measure the reflectance in a region of dense fog. He et al. proposed a method of estimating the transmission map using the dark channel prior (DCP) [7]. The DCP theory is based on the observation that the minimum intensity of one of the RGB channels in a fog-free region is close to zero. However, it cannot avoid color distortion, since the transmission map is estimated using the color of the object. In addition, in order to remove the blocking and the halo effects appearing in the process of estimating the transmission map, a computationally expensive soft-matting algorithm is used. To solve this problem, Gibson et al. replaced the soft-matting step with a standard median filter [8], Xiao et al. removed the blocking and halo effects using a joint bilateral filtering [9], Chen et al. used a gain intervention refinement filter [10], and Jha et al. used an $l_{2}$ norm prior [11]. Yoon et al. proposed a defogging method using the multiphase level set in the HSV color space and corrected colors between adjacent video frames [12]. Meng et al. proposed a method to estimate the transmission map using a boundary constraint, and they refined the transmission map through $l_{1}$ norm-based regularization [13]. Ancuti et al. proposed a multiscale fusion-based defogging algorithm using a Laplacian pyramid and a Gaussian pyramid, both of which improved a single foggy image using white balance and contrast enhancement, respectively [14]. Berman et al. proposed a nonlocal-based defogging algorithm using an estimated transmission based on color lines [15]. However, these methods are not free from color distortion, since they do not consider the depth information. Recently, several learning-based defogging methods have been proposed [16,17,18,19]. Chen et al. proposed a radial basis function (RBF) network to restore a foggy image while recovering visible edges [16]. Cai et al. proposed a trainable end-to-end system to estimate the medium transmission, called DehazeNet [17]. Eigen et al. proposed a convolutional neural network (CNN) architecture to remove raindrop and lens dirt [19].

Many defogging methods were proposed using the depth information. Caraffa et al. proposed a depth-based defogging method using Markov random field model to generate the disparity map using a stereo image pair [20]. Lee et al. estimated the scattering coefficient of the atmosphere in the stereo image [21]. Park et al. estimated the depth in the stereo image pair and removed the fog by estimating the atmospheric light in the farthest region [22]. However, accurate estimation of the transmission map is still an open problem, since the features for obtaining the disparity map are generally distorted in the foggy image.

To improve the problem of existing single-image-based defogging algorithms, this paper presents a novel image defogging algorithm using a stereo foggy image pair. The proposed defogging algorithm removes fog by estimating the depth information from the stereo image pair and iteratively improving the depth information. The disparity of an input stereo foggy image pair is first obtained using the optical flow, and the depth map is generated using the disparity. Next, the transmission map is estimated using the generated depth map to remove the foggy component. The optical flow and transmission map estimation steps repeat until the defogged solution converges. The proposed stereo-based defogging algorithm is suitable for dual cameras embedded in high-end smartphone models that were recently released on the consumer market.

The paper is organized as follows: Section 2 describes a physical degradation model for foggy image acquisition, and Section 3 presents the proposed stereo-based defogging algorithm based on the degradation model. Section 4 summarizes experimental results, Section 5 presents an application of the proposed defogging algorithm to an asymmetric dual camera system, and Section 6 concludes the paper.

## 2. Physical Degradation Model of Foggy Image Acquisition

**Figure 1:**
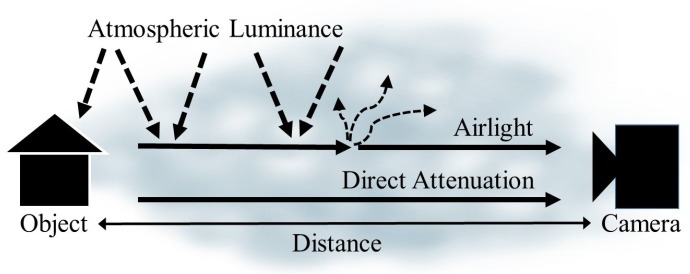
Illustration of the foggy image formation model.

Figure 1 shows the physical degradation model of foggy image acquisition. The light reflected by the object is absorbed and scattered by fog particles in the atmosphere, and arrives at the camera sensor. Therefore, the greater the distance between the object and the camera is, the greater the atmospheric degradation becomes. The foggy image *g* is defined according to the Koschmieder model [23] as

(1) g(x,y)=f(x,y)t(x,y)+A(1−t(x,y)),

where (x,y) represents the pixel coordinate, f(x,y) the fog-free image, and the constant *A* the global atmospheric light. t(x,y) represents the transmission coefficient at pixel (x,y), and can be expressed as

(2) $$t\left(x,y\right)=e^{-\beta \cdot d(x,y)},$$

where β represents the scattering coefficient of the atmosphere, and d(x,y) the depth between the scene point and the camera. From (1) , an intuitive estimation of the fog-free image is given as

(3) $$\hat{f}\left(x,y\right)=\left(g\left(x,y\right)-A\right)/\max\left(t\left(x,y\right),t_{0}\right)+A,$$

The defogged image $\hat{f}\left(x,y\right)$ is obtained by substituting the estimated *A* and t(x,y) into (3). $t_{0}$ is the lower bound of t(x,y), which is set to an arbitrary value to avoid the zero in the denominator.

## 3. Image Defogging Based on Iteratively Refined Transmission

**Figure 2:**
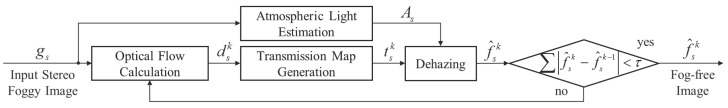
Block diagram of the proposed defogging algorithm: For s∈{L,R}, $g_{s}$ is the input stereo image pair, $A_{s}$ is the global atmospheric light, $d_{s}^{k}$, $t_{s}^{k}$, and ${\hat{f}}_{s}^{k}$, respectively, are the disparity map, the transmission map, and the defogged image at the *k*-th iteration.

Most existing defogging algorithms estimate the disparity map from the stereo foggy image pair, and then obtain the defogged image by estimating the transmission map using (2). However, it is difficult to detect the feature to estimate the disparity map, since the foggy image is distorted by the fog component. To solve this problem, the proposed algorithm estimates an accurate transmission map by iteratively improving the disparity map. The disparity map is generated by estimating optical flow from the stereo foggy image pair, and the initial transmission map is generated by the disparity. Atmospheric light *A* is estimated using the color line theory [24], and each stereo foggy image is restored using (3). By repeating the set of optical flow estimation, transmission map generation and defogging steps, a progressively improved transmission map and better defogged image are obtained. This process repeats until the absolute difference between the *k*th and (k−1)th defogged images is less than a pre-specified threshold τ. Figure 2 shows the block diagram of the proposed algorithm.

### 3.1. Atmospheric Light Estimation

Most single-image-based defogging algorithms set the atmospheric light *A* to an arbitrary constant or to the brightest pixel value in the image under the assumption that the fog color is white [5,6,7]. Since these methods do not estimate the accurate atmospheric light, the quality of defogged images is degraded. In this paper, the atmospheric light *A* is estimated using the color line-based estimation method that was originally proposed by Sulami et al. [25].

In (1), the fog-free image f(x,y) can be expressed as

(4) f(x,y)=l(x,y)R(x,y),

where surface shading l(x,y) is a scalar value indicating the magnitude of the reflected light, and surface albedo R(x,y) is an RGB vector representing the chromaticity of the reflected light. In general, when a natural image is divided into small patches, the surface albedo and transmission of each image patch are approximately constant. Therefore, using this characteristic and (4), the foggy image formation model in (1) can be expressed as follows:

(5) $$g\left(x,y\right)=t_{i}\left(x,y\right)l\left(x,y\right)R_{i}\left(x,y\right)+A\left(1-t_{i}\right),$$

where $t_{i}\left(x,y\right)$ represents the transmission value of the *i*-th image patch, R(x,y) the surface albedo of the patch. To create color lines in the RGB color space using image patches with the same surface albedo and transmission, image patches satisfying (5) are selected using principal component analysis (PCA). The strongest principal axis of the image patch should correspond to the orientation of the color line, and there should be a single significant principal component. Additionally, the color line of the image patch should not pass the origin in RGB space, and the image patch should not contain an edge. The color lines are generated using image patches that satisfy these conditions, and the orientation and magnitude of vector *A* is estimated.

### 3.2. Stereo Image Defogging

To generate the transmission of a stereo image pair, the disparity map is estimated using the combined local–global approach with total variation (CLG–TV) [26]. The CLG–TV approach integrates Lucas–Kanade [27] and Horn–Schunck [28] models to estimate motion boundary-preserved optical flow using a variational method. The $\ell_{1}$ norm error function of the Horn–Schunck model is defined as

(6) $$E_{HS}=\int_{\Omega }\left[\lambda r{\left(u,v\right)}^{2}+\left({\left|\nabla u\right|}^{2}+{\left|\nabla v\right|}^{2}\right)\right],$$

where r(u,v) represents the residual between the left and right images as

(7) $$r\left(u,v\right)=g_{R}\left(x+u_{0}\right)-g_{L}\left(x\right)+{\left(u-u_{0}\right)}^{T}\nabla g_{R}\left(x+u_{0}\right)≅0\;,$$

where $u=\left(u,v\right):\Omega \to R^{2}$ is the optical flow to estimate with the initial value $u_{0}$, and the left and right images are respectively given as

(8) $$g_{L}\left(x\right)=g_{R}\left(x+u\right)=g_{R}\left(x+u_{0}+u-u_{0}\right)≅g_{R}\left(x+u_{0}\right)+{\left(u-u_{0}\right)}^{T}\nabla g_{R}\left(x+u_{0}\right)\;,$$

where $x=\left(x,y\right)\in \Omega \subset R^{2}$, $E_{HS}$ can be minimized by solving the Euler–Lagrange equation using Jacobi iteration. To make the estimated optical flow as uniform as possible in a small region, the residual r(u,v) in (6) is substituted by the Lucas–Kanade error function

(9) $$E_{LK}=\sum_{window}w\cdot r{\left(u,v\right)}^{2},$$

where *w* represents the weighting factor. $E_{LK}$ is minimized solving a least-squares problem. Based on the Lucas–Kanade model, the total error of the same window is minimized. The following error function combines Horn–Schunck and Lucas–Kanade models.

(10) $$E_{CLG-HS}=\int_{\Omega }\left[\lambda \left(\sum_{window}r{\left(u,v\right)}^{2}\right)+\left({\left|\nabla u\right|}^{2}+{\left|\nabla v\right|}^{2}\right)\right]$$

To solve the over-smoothing problem in motion boundaries regions, the $\ell_{1}$ norm is minimized instead of the $\ell_{2}$ norm.

(11) $$E_{CLG-TV}=\int_{\Omega }\left[\lambda \left(\sum_{window}r{\left(u,v\right)}^{2}\right)+\left(\left|\nabla u\right|+\left|\nabla v\right|\right)\right]$$

To minimize the CLG–TV error function, we use an alternative Horn–Schunck model [29] where $E_{CLG-TV}$ is decomposed into three terms, as shown below.

(12) $$E_{CLG-TV}=\int_{\Omega }\left[\lambda \left(\sum_{window}r{\left(u,v\right)}^{2}\right)+\frac{1}{2\beta }{\left(u-\hat{u}\right)}^{2}+\frac{1}{2\beta }{\left(v-\hat{v}\right)}^{2}\right]$$

(13) $$E_{TV-u}=\int_{\Omega }\left[\frac{1}{2\beta }{\left(u-\hat{u}\right)}^{2}+\left|\nabla u\right|\right]$$

(14) $$E_{TV-v}=\int_{\Omega }\left[\frac{1}{2\beta }{\left(v-\hat{v}\right)}^{2}+\left|\nabla v\right|\right]$$

$E_{CLG-TV}$ is minimized in the point-wise manner, whereas $E_{TV-u}$ and $E_{TV-v}$ are minimized using the procedure proposed by Chambolle [30]. In this paper, we calculated the disparity map for only *u* from the stereo image.

**Figure 3:**
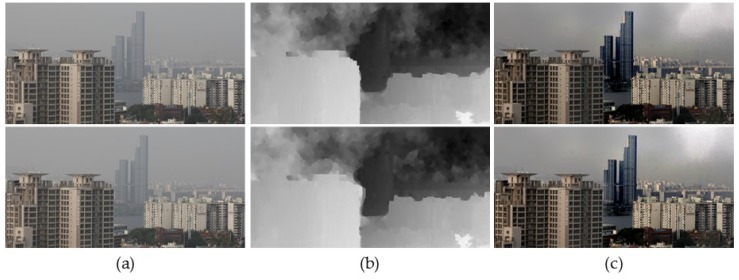
Results of defogging using the initial transmission map. (**a**) A stereo pair of input foggy images; (**b**) initial transmission maps; and (**c**) defogged results.

Figure 3 shows the result of the transmission map estimation and fog removal using a stereo input image pair. Since the disparity map is generated using the distorted features by foggy component, the initially estimated transmission map is not sufficiently clear.

### 3.3. Iterative Refinement of Transmission Map

In this subsection, an iterative process is performed to refine the transmission map. The disparity map is estimated again on the defogged image ${\hat{f}}_{s}\left(x,y\right)$, estimated by using the initial transmission map $t_{s}\left(x,y\right)$, and the transmission map is updated. The *k*th defogged image ${\hat{f}}_{s}^{k}\left(x,y\right)$ is obtained by the updated transmission map $t_{s}^{k}\left(x,y\right)$.

**Figure 4:**
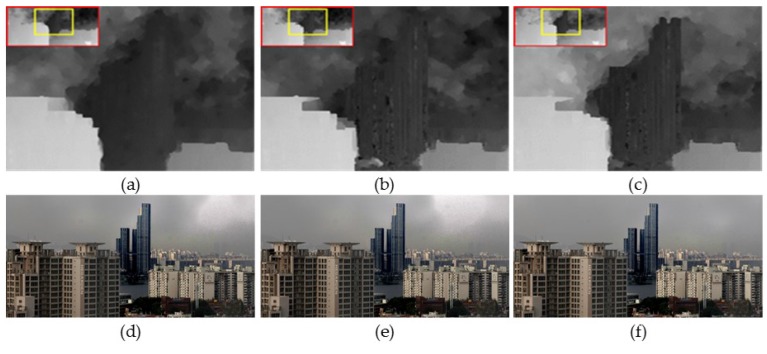
Iterative refinement process of transmission map. (**a**–**c**) The 1-st, 3-rd, and 5-th refined transmission maps; and (**d**–**f**) the 1-st, 3-rd, and 5-th defogged results.

Figure 4 shows the estimated transmission map and the result of fog removal through the iterative process. As shown in Figure 4, color distortion in the sky region is gradually reduced.

**Figure 5:**
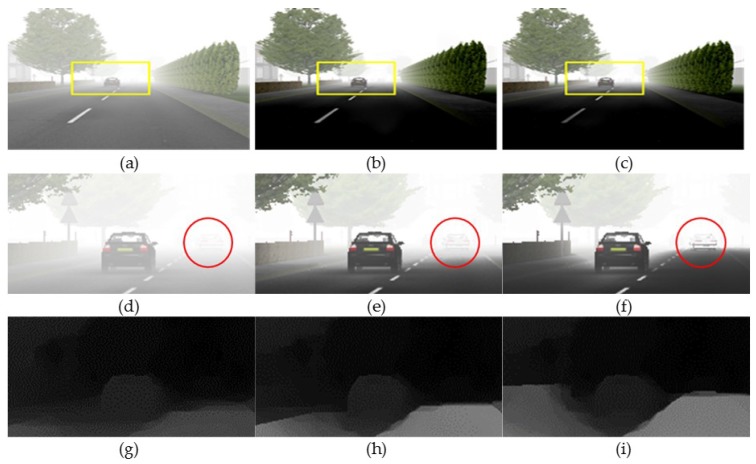
Defogging results of synthetic stereo image. (**a**–**c**) Input, 4-, and 7-th defogged images; (**d**–**f**) enlarged version of (a–c); and (**g**–**i**) corresponding transmission maps.

Figure 5 shows the iteratively refined transmission maps and the correspondingly defogged results using the FRIDA3 dataset (Foggy Road Image DAtabase) [20]. As the fog is removed in the distant region, the transmission map is gradually improved in the iterative process. As a result, the red circle region is iteratively improved and the initially invisible vehicle appears.

**Figure 6:**
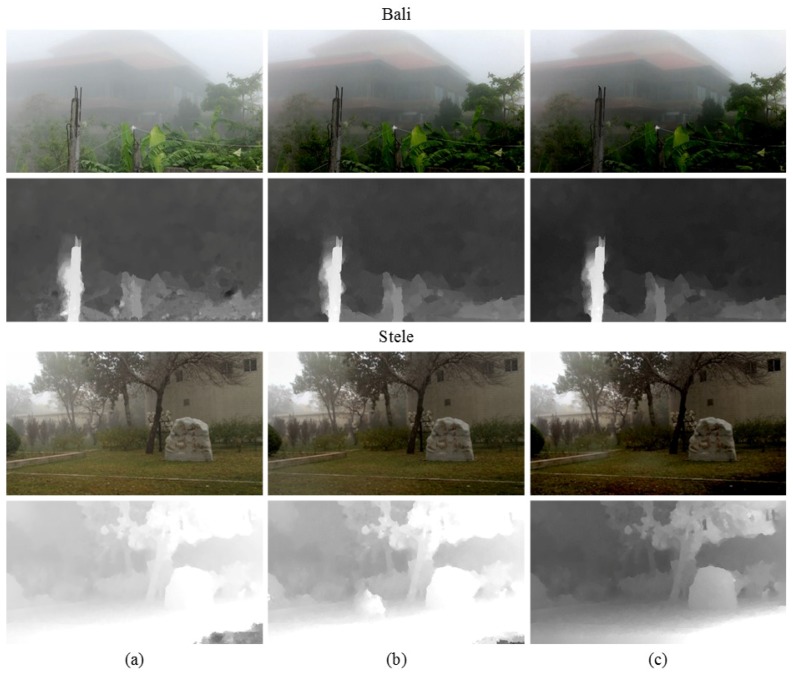
Defogging results using real-world videos from [31]. (**a**) Input foggy images; (**b**) defogged results after three iterations; and (**c**) defogged results after five iterations. Corresponding transmission maps are shown below each image.

Figure 6 shows the iteratively defogged results using real-world videos [31]. We extracted two adjacent frames in a video and assumed a situation of acquiring images using a dual camera. In the iteration process, the transmission map is gradually refined, and the defogged result is improved.

## 4. Experimental Results

**Figure 7:**
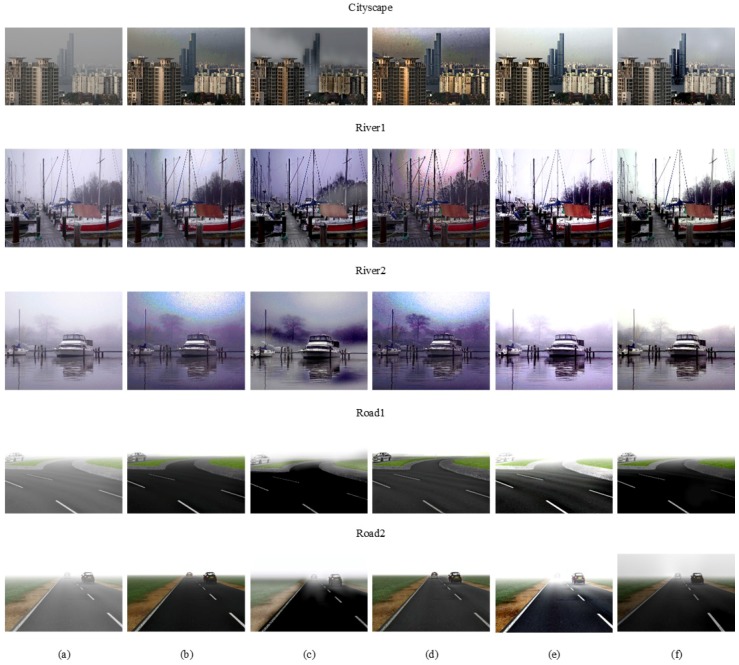
Experimental results of various defogging methods. (**a**) Input foggy images; (**b**) He et al. [7]; (**c**) Ancuti et al. [14]; (**d**) Meng et al. [13]; (**e**) Berman et al. [15]; (**f**) the proposed method.

To evaluate the performance of the proposed defogging method, experimental results are compared with those of the state-of-the-art defogging algorithms. Figure 7a shows a set of test foggy images, Figure 7b–f respectively shows results of He’s method [7], Ancuti’s method [14], Meng’s method [13], Berman’s method [15], and the proposed method.

In Cityscape, River1, and River2 results, Figure 7b,d shows that color distortion and low saturation artifacts occur in the sky region because the atmospheric light is not accurately estimated. Figure 7c shows that the color of the sky region is distorted and the color around the building is faded because the same amount of fog is removed without considering spatially different depth information. Figure 7e shows a slight amount of color distortion since the initial transmission map is regularized by using Gaussian Markov random fields with only local neighbors. Figure 7f shows that the defogged result is clearer than any other method in the sky region, and the color contrast is increased.

In Road1 and Road2 results, Figure 7c shows that the color around the road is faded and distorted because it does not consider depth information. Figure 7e shows that the color tends to be oversaturated when the atmosphere light is significantly brighter than the scene. Figure 7b,d shows excellent defogging results because the color of artificially added fog is mostly white, so the transmission map is well estimated by the DCP-based defogging algorithm. Figure 7f shows a well-defogged result without color distortion or saturation. Experimental results demonstrated that the proposed algorithm outperforms existing algorithms in terms of both fog removal and color preservation.

Table 1 shows two quantitative measures for objective evaluation, including no-reference image quality metric for contrast distortion (NIQMC) [32] and entropy for measuring contrast of the defogged results. The higher NIQMC value indicates superior color contrast and edge of the image. A high entropy value indicates that the average amount of information in the image is high. In other words, a greater amount of information about the edges or features results in better color contrast. Based on Table 1, the highest values in each image are shown in bold and the proposed method performs better than other existing methods.

## 5. Application to Asymmetric Dual Camera System

**Figure 8:**
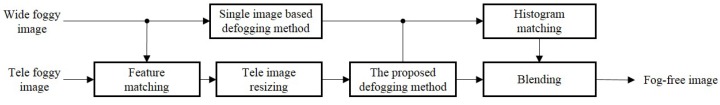
Block diagram of the proposed defogging algorithm applied to an asymmetric dual camera system.

The proposed stereo-based defogging algorithm is particularly suitable for a dual camera system that has attracted increasing attention in the field of robot vision, autonomous driving, and high-end smartphones. Figure 8 shows the block diagram of the proposed defogging algorithm applied to an asymmetric dual camera system. To estimate the depth information, features in the stereo foggy image pair are first matched, and the scale of the longer focal length image is then corrected. The proposed defogging method is applied to the overlapped regions of two images with different focal lengths. To remove the fog in the non-overlapping region, a single image-based defogging method is first used, and color distortion is then corrected using histogram matching with reference to the overlapped region.

**Figure 9:**
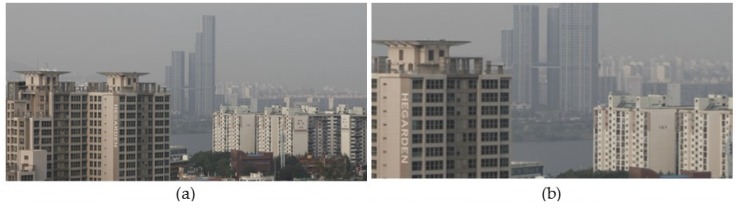

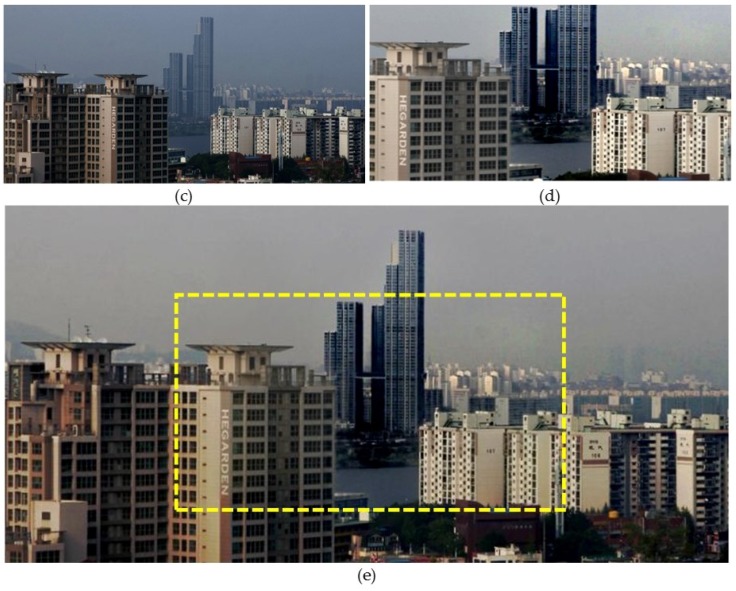
Defogging results using a simulated pair of dual camera images. (**a**) An input foggy image; (**b**) simulated longer focal length; (**c**) defogged version of (a) using a single-image-based method; (**d**) defogged version of (b) using the proposed stereo-based method; (**e**) stitched result of (c,d).

Figure 9 demonstrates that the proposed algorithm can be applied to an asymmetric dual camera system. For the experiment, Figure 9b is obtained by cropping the center region of Figure 9a. As a result, Figure 9a,b can be considered as a stereo pair of input foggy images with different focal lengths. Figure 9c shows the defogged result of Figure 9a by the single image defogging method. Figure 9d shows the defogged result of Figure 9b by the stereo image defogging method proposed in this paper. Figure 9e shows the stitching result of Figure 9c,d. Based on the result, the proposed stereo-based method can remove fog while preserving the original color information in the asymmetric dual camera system.

## 6. Conclusions

In this paper, a stereo defogging algorithm is proposed to accurately estimate the transmission map based on the depth information. The major contribution of this work is twofold: (i) The stereo-based iterative defogging process can provide greatly enhanced results compared with existing state-of-the-art methods; and (ii) the framework of the stereo-based algorithm is particularly suitable for a dual camera system that is embedded in a high-end consumer smartphone. The proposed method first obtains the disparity map by estimating optical flow from the stereo foggy image pair, and generates the initial transmission map using the disparity. The defogged image is restored by generating the transmission map and estimating atmospheric light *A* based on the color line theory. By repeating the set of optical flow estimation, transmission map generation, and defogging until convergence, significantly improved defogged results were obtained. Experimental results show that the proposed method successfully removed fog without color distortion, and the transmission map and the defogged image were iteratively refined. The proposed method can be used as a pre-processing step for an outdoor image analysis function in an intelligent video surveillance system, autonomous driving, and asymmetric dual camera in a high-end smartphone.

## Figures and Tables

**Table 1:** Quantitative results for each method using stereo images. NIQMC: no-reference image quality metric for contrast distortion.

	Foggy Image	Cityscape	River1	River2	Road1	Road2	Average
NIQMC	He et al. [7]	5.1098	**5.6812**	5.3248	4.3460	4.0199	4.8963
	Ancuti et al. [14]	4.8153	5.2894	5.2402	4.3295	4.4500	4.8248
	Meng et al. [13]	**5.3644**	5.6608	5.2500	3.9786	3.7448	4.7997
	Berman et al. [15]	4.9471	5.0327	5.4835	**4.4345**	4.8695	4.9534
	Proposed method	4.9757	5.1463	**5.6014**	4.1673	**4.9004**	**4.9582**
Entropy	He et al. [7]	7.2919	7.7846	7.4451	4.2163	4.2776	6.2031
	Ancuti et al. [14]	7.1363	7.4826	7.4117	3.0374	4.0065	5.9594
	Meng et al. [13]	**7.5326**	**7.6925**	7.5026	4.1155	4.1647	6.2015
	Berman et al. [15]	7.6657	6.7250	6.6159	4.4345	4.4563	5.9594
	Proposed method	7.1546	7.2941	**7.7196**	**4.5470**	**5.5631**	**6.4556**
